# Accurate interpretation of p53 immunohistochemical patterns is a surrogate biomarker for *TP53* alterations in large B-cell lymphoma

**DOI:** 10.1186/s12885-023-11513-x

**Published:** 2023-10-19

**Authors:** Xinyi Li, Danju Luo, Liling Zhang, Qiuhui Li, Jun Fan, Jiwei Zhang, Bo Huang, Ming Yang, Xiu Nie, Xiaona Chang, Huaxiong Pan

**Affiliations:** 1 Department of Pathology, Union Hospital, Tongji Medical College, Huazhong University of Science and Technology, Wuhan, 430022 China; 2 Cancer Center, Union Hospital, Union Hospital, Tongji Medical College, Huazhong University of Science and Technology, Wuhan, 430022 China

**Keywords:** Lymphoma, Large B-cell lymphoma, p53 immunohistochemistry, Surrogate marker, *TP53* alterations

## Abstract

**Background:**

To clarify the relationship between p53 immunohistochemistry (IHC) staining and *TP53* alterations (including mutations and deletions) in large B-cell lymphomas (LBCLs) and to explore the possibility of p53 IHC expression patterns as surrogate markers for *TP53* alterations.

**Methods:**

A total of 95 patients diagnosed with LBCLs were selected, and paraffin samples were taken for *TP53* gene sequencing, fluorescence in situ hybridization and p53 IHC staining. The results were interpreted by experienced pathologists and molecular pathologists.

**Results:**

Forty-three nonsynonymous *TP53* mutations and p53 deletions were detected in 40 cases, whereas the remaining 55 cases had wild-type *TP53* genes. The majority of *TP53* mutations (34/43, 79.1%) occurred in exons 4-8, and R248Q was the most common mutation codon (4/43, 9.3%). The highest frequency single nucleotide variant was C > T (43.6%). p53 expression was interpreted as follows: Pattern A: p53 staining was positive in 0%-3% of tumor cells, Pattern B: p53 staining was positive in 4-65% of tumor cells, Pattern C: more than 65% of tumor cells were stained positive for p53. The p53 IHC expression patterns were associated with *TP53* alterations. Gain of function variants and wild-type *TP53* tended to exhibit type C and B p53 expression patterns, but loss of function variants were exclusively seen in type A cases. Additionally, interpretation of the staining by various observers produced significant reproducibility.

**Conclusions:**

The p53 IHC expression patterns can be used to predict *TP53* alterations and are reliable for diverse alteration types, making them possible surrogate biomarkers for *TP53* alterations in LBCLs.

**Supplementary Information:**

The online version contains supplementary material available at 10.1186/s12885-023-11513-x.

## Background

According to the conceptual framework in lymphoid neoplasms in the 5th edition of the WHO Classification of Haematolymphoid Tumors, the large B-cell lymphoma (LBCL) family comprises a wide spectrum of tumors. LBCLs include 18 specific entities, of which diffuse large B-cell lymphoma, not otherwise specified (DLBCL, NOS), represents the most common entity [1].

*TP53*, as the most frequently disrupted gene in malignant diseases, participates in many biological processes, such as DNA repair, cell cycle arrest, apoptosis, and autophagy [2–4]. *TP53* mutations are present in 25-50% of sporadic B-cell lymphomas and approximately 20% of DLBCLs at diagnosis [5, 6], which are associated with adverse clinical outcomes in LBCLs and other lymphoid neoplasms [6–10]. Meanwhile, *TP53* mutations are involved in chemoresistance and progression of cancer and are increased upon the recurrence of DLBCLs and other lymphatic malignancies [11–14]. Therefore, the detection of *TP53* mutations is of considerable importance in the clinical practice of patients with LBCLs.

Although nucleotide sequencing and FISH are the most reliable techniques for detecting gene mutations and chromosomal deletions, it is not routinely used in pathological diagnosis, because of its complexity and high cost. In contrast, immunohistochemistry (IHC) techniques, which are routinely used for pathological diagnosis, are much simpler, inexpensive and faster. p53 is a 393-amino acid protein encoded by the *TP53* gene [15], whose protein structure is usually altered in response to mutations in *TP53*, further leading to changes in its expression pattern [16, 17]. The gain of function (GOF) effect of the *TP53* gene is usually induced by missense mutations and in-frame indels, resulting in a strong positive p53 staining pattern. The loss of function (LOF) effect caused by nonsense mutations, splicing mutations, and frameshift indels is usually manifested by a weak expression pattern of p53 [15]. Additionally, the loss of the *TP53* gene can lead to the loss of *TP53* function, which is also considered as one type of *TP53* LOF effect [18]. The p53 IHC expression patterns have been proven to be surrogate markers for *TP53* alterations (including mutations and deletions [19]) in several types of cancer [15, 20]. However, it is less studied whether p53 expression patterns are reliable predictors and surrogate markers of *TP53* alterations in LBCLs [21–25].

In the present study, we analyzed the relationship between p53 IHC expression patterns and *TP53* alterations in LBCLs to clarify whether p53 IHC expression patterns can be used as predictors of *TP53* alterations.

## Methods

### Patients and tissues

By searching for surgical resection or biopsy specimens with a pathological diagnosis of LBCLs (according to the 5th edition of the World Health Organization classification of hematolymphoid tumors [1], Table S1) in Union Hospital, Tongji Medical College, Huazhong University of Science and Technology from 2019 to 2022, candidates were retrospectively selected from the institutional database of the center. HE slides were reviewed by two pathologists to reach a consensus diagnosis of LBCLs. Clinicopathological features, including age, sex, primary site, histological type, double expression (overexpression of both C-MYC protein (positivity rate > 40%) and BCL2 protein (positivity rate > 50%)), Ki67 percentage score, cell origin, and stage, were recorded. Cases with insufficient specimens for mutational analysis (tumor cells in the tissue sections < 20%) were excluded. The study was conducted in accordance with the principles outlined in the Declaration of Helsinki. The study was approved by the Medical Ethics Committee of Tongji Medical College, Huazhong University of Science and Technology (2018-S377). Written informed consent was obtained from individual or guardian participants.

### DNA extraction, sequencing, and bioinformatics analysis

Using the FFPE DNA LQ kit, genomic DNA was extracted from formalin fixed paraffin-embedded (FFPE) tumor tissues. This extraction process involved meticulous tissue preparation, deparaffinization, and proteinase K digestion. The purified DNA was then quantified using NanoDropOne (ThermoFisher) microvolume spectrophotometer to ensure sufficient quality and quantity for downstream analysis. The extracted DNA underwent a series of preparatory steps to facilitate high-throughput sequencing. The KAPA DNA HyperPlus Kit (KAPA Biosystems) was employed for DNA preparation. This process included several steps: DNA fragmentation to produce appropriately sized fragments, followed by end repair and the ligation of adapters containing uniquely identifying sequences. These steps were crucial for making the DNA compatible with the sequencer and for distinguish each sample. The constructed genomic library underwent hybridization capture to enrich the target regions for targeted sequencing. This enrichment process involved the use of customized probes (IDT) and the xGen™ NGS Hybridization Capture kit (IDT). The libraries were sequenced on the Illumina high-throughput sequencing NextSeq 550Dx platform (Illumina, CA, USA) to generate 150-bp paired-end reads. This sequencing technology allowed for high-throughput data generation and increased coverage of the *TP53* gene, ensuring comprehensive mutation analysis. Point mutations and short fragment insertion/deletion mutations in the *TP53* protein coding region and paratenic intron region were analyzed in comparison with the GRCh37/hg19 reference genome assembly. To enhance the accuracy of variant identification, a multi-step approach was employed. Variant data were cross-referenced with established public databases, including dbSNP, 1000Genomes, gnomAD, COSMIC, and ClinVar, to verify their presence in the population. Additionally, functional prediction software tools such as Polyphen2 and SIFT were employed to assess the potential impact of detected variants on p53 protein function. A variant allele frequency (VAF) cut-off of 1% was used to exclude false positive (artefact and germline polymorphisms) variants within the cohorts.

### *TP53* fluorescence in situ hybridization (FISH) staining

FFPE tissue sections underwent standard deparaffinization and dehydration protocols to ensure optimal tissue quality for subsequent analysis. The FISH probe (FP-014-2, Wuhan HealthCare Biotechnology Co., Ltd) used in this study was specifically designed to target the *TP53* gene locus. The P53/CEP17 dual-color probe employs orange fluorescence-labeled probes to target the *TP53* gene region and green fluorescence-labeled probes to target the centromere region of chromosome 17. The *TP53* gene region is targeted using the BAC clone RP11-625M17, which has been verified through the UCSC website to fully cover the *TP53* gene within the interval chr17:7,542,877-7,727,896 (GRCh38/hg38), spanning a length of 185kb. The CEP17 probe utilizes specific α-satellite sequences from the centromere region of chromosome 17 for labeling. FISH analysis was conducted following the manufacturer's instructions. Briefly, tissue sections were subjected to heat pretreatment for target retrieval and then denatured. The FISH probe was applied to the tissue sections, and hybridization was allowed to occur at the appropriate temperature (denaturation 85 °C for 5 min, annealing 42 °C for 16 h). After hybridization, the slides were washed to remove any unbound probe. Cell nuclei were counterstained with 4',6-diamidino-2-phenylindole (DAPI). The slides were then examined under a fluorescence microscope equipped with appropriate filters for detecting the FISH signals. Images were captured and analyzed using Metasystem. For each sample, at least 100 non-overlapping nuclei were analyzed to determine the *TP53* gene status. The presence or absence of *TP53* gene aberrations, such as deletions or amplifications, was recorded. The FISH results were classified as either normal (no *TP53* gene aberrations) or abnormal (presence of *TP53* gene aberrations).

### p53 IHC staining

IHC staining was performed using a Ventana Benchmark Ultra platform (Roche, Basel, Switzerland) with a p53 monoclonal antibody, DO-7 (Roche, Switzerland). The visualization system employed was the OptiView DAB IHC detection kit (06396500001, Roche, Switzerland). Specifically, paraffin-embedded tissues were sectioned to a thickness of 3 microns, baked at 65 °C for 1 h, and then processed using an automated immunohistochemistry instrument. The detection conditions included CC1 repair solution for 64 min at 95 °C, with primary antibody incubation for 36 min, universal linker for 12 min, secondary antibody for 12 min, and DAB for 8 min, all at 37 °C.

The percentage of p53-positive cells (p53 percentage-score) was calculated based on the following criteria: Cells with a signal in the nucleus were considered p53-positive, and the percentage of positive cells was then quantified and scored on a scale ranging from 0 to 100%.

p53 IHC score (H-score) was calculated based on the following criteria: Firstly, p53 expression was categorized into three intensity levels: strong, moderate, and weak, with assigned values of 3, 2, and 1, respectively. Subsequently, the proportions of staining intensity for strong, moderate, and weak staining were quantified as percentages (ranging from 0 to 100%), and corresponding scores of 0 to 100 were assigned to each proportion. The H-score for each case was determined by multiplying the score of staining intensity by its corresponding proportion and summing the values, resulting in a range from 0 to 300 for the H-score.

### Statistical analysis

All statistical analyses were performed using SPSS version 26.0 (IBM Corp., Armonk, NY, USA). The dataset was managed and prepared for analysis using standard data cleaning and preprocessing techniques, including data validation, removal of outliers, and handling of missing data as applicable. To investigate the relationships between the p53 IHC patterns and *TP53* alterations, appropriate statistical tests were applied. Specifically: The chi-square (χ2) test was employed to assess associations between categorical variables, such as different p53 IHC patterns and *TP53* alterations. This test determined whether observed associations were statistically significant. In cases where the χ2 test assumptions were not met (e.g., small sample sizes), Fisher’s exact test, a robust alternative, was used to examine associations between categorical variables. The receiver operating characteristic (ROC) curve analysis was utilized to assess the predictive performance of the identified p53 percentage-score and p53 H-score in distinguishing different types of *TP53* alterations. The Youden index was calculated to determine the optimal detection threshold for the p53 percentage-score and the p53 H-score. Diagnostic tests (sensitivity, specificity, and overall accuracy) were applied to assess the performance of the p53 percentage-score and p53 H-score in predicting *TP53* alterations. The Kappa identity test and Pearson correlation analysis were used to evaluate the consistency and correlation between different observers. A two-sided *p*-value < 0.05 was considered statistically significant.

## Results

### Patient population

Among 95 patients, *TP53* wild-type (comfirmed by next generation sequencing (NGS) and FISH analysis) was detected in 55 cases (57.9%), and *TP53* alterations (including 43 nonsynonymous *TP53* mutations and p53 deletions, comfirmed by NGS and FISH) were detected in 40 cases (42.1%). There were 50 males (52.6%) and 45 females (47.4%). Ninety-five cases aged 5 to 81 (median age 56). Seventy-five patients (78.9%) were diagnosed with DLBCL, and 20 patients (21.1%) were diagnosed with other types of LBCL. There were 10 (10.5%) patients with stage I disease, 18 (18.9%) patients with stage II disease, 8 (8.4%) patients with stage III disease, 49 (51.6%) patients with stage IV disease, and 10 (10.5%) patients who did not require or were difficult to stage. There were 5 (5.3%) patients with Ki67 positivity ≤ 30%, 42 (44.2%) patients with Ki67 positivity between 31 and 70%, and 48 (50.5%) patients with Ki67 positivity > 70%. Except for age, there was no significant difference in other characteristics between the mutant/delete-type group and the wild-type group. The mutant/delete group was younger, with an average age of 48 years, which was lower than that of the wild-type group (55 years) (Table 1).

**Table 1:** Clinicopathological characteristics of 95 LBCLs

Characteristics	Alteration status		Total number n%	*p*-value
	Wild type (*n* = 55)	Mutant/delete type (*n* = 40)		
Gender				0.100
male	25 (45.5)	25 (62.5)	50 (52.6)	
female	30 (54.5)	15 (37.5)	45 (47.4)	
Age (year)				0.028
≤ 35	5 (9.1)	11 (27.5)	16 (16.8)	
36—45	9 (16.4)	1 (2.5)	10 (10.5)	
46—55	9 (16.4)	10 (25.0)	19 (20.0)	
56—65	15 (27.3)	10 (25.0)	25 (26.3)	
> 65	17 (30.9)	8 (20.0)	25 (26.3)	
Tumor location				0.072
Nodal lymphoma	33 (60.0)	31 (77.5)	64 (67.4)	
Intranodal lymphoma	22 (40.0)	9 (22.5)	31 (32.6)	
Histological type				0.830
Others	12 (21.8)	8 (20.0)	20 (21.1)	
DLBCL	43 (78.2)	32 (80.0)	75 (78.9)	
Double expressors				0.212
Not applicable	2 (3.6)	5 (12.5)	7 (7.4)	
No	16 (29.1)	8 (20.0)	24 (25.3)	
Yes	37 (67.3)	27 (67.5)	64 (67.4)	
Cell of origin				0.225
Not applicable	2 (3.6)	5 (12.5)	7 (7.4)	
ABC subtype	35 (63.6)	21 (52.5)	56 (58.9)	
GCB sybtype	18 (32.7)	14 (35.0)	32 (33.7)	
Stage				0.348
Not applicable/Difficult to determine	3 (5.5)	7 (17.5)	10 (10.5)	
I	7 (12.7)	3 (7.5)	10 (10.5)	
II	12 (21.8)	6 (15.0)	18 (18.9)	
III	4 (7.3)	4 (10.0)	8 (8.4)	
IV	29 (52.7)	20 (50.0)	49 (51.6)	
Ki67				0.288
≤ 30	2 (3.6)	3 (7.5)	5 (5.3)	
31—70	28 (50.9)	14 (35.0)	42 (44.2)	
> 70	25 (45.5)	23 (57.5)	48 (50.5)	

*ABC* activated B-cell-like, *GCB* germinal center B-cell-like

### Analysis of *TP53* mutations

**Fig. 1:**
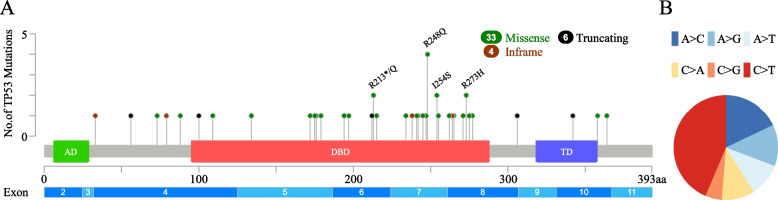
**A**. The “lollipop” plot of the *TP53* gene, as well as the frequency and position of *TP53* mutations in LBCLs in our study. **B**. Percentage of the 6 possible mutation classes. AD: transactivation domain; DBD: DNA binding domain; TD: tetramerization domain

As shown in Fig. 1A, among 95 LBCL cases, 43 nonsynonymous *TP53* mutations were detected via NGS in 40 cases. Most of the 43 *TP53* mutations occurred in exons 4-8 (34/43, 79.1%), and exon 7 (13/43, 30.2%) was the most common mutated exon. Most of the mutations were located in the DNA-binding domain, and the most frequent mutation site was R248Q (4/43, 9.3%). In the statistical analysis of single nucleotide variants, we found that the highest frequency was C > T (43.6%), followed by A > C (17.9%), A > G (12.8%), A > T (10.3%), C > A (10.3%), and C > G (5.1%) (Fig. 1B).

### P53 Immunohistochemistry

ROC analysis was performed to assess the discriminative value of p53 percentage-score for *TP53* LOF variants, wild-type, and GOF variants in LBCLs. Among the 95 cases, two cases with both LOF and GOF variants were excluded for ROC curve plotting. Initially, the 93 cases were divided into the LOF variant group (9 cases) and non-LOF variant group (84 cases), and the ROC curve was plotted (Figure S1A). The results of ROC analysis revealed that p53 percentage-score could serve as a valuable biomarker to distinguish *TP53* LOF variants from non-LOF variants (area under curve, AUC = 0.917, *p* < 0.001). The Youden Index reached its maximum at p53 percentage-score = 3.5 (Youden Index = 0.817), with corresponding sensitivity and specificity of 0.929 and 0.889, respectively (Figure S1A). Subsequently, the 93 cases were divided into the non-GOF variant group (64 cases) and GOF variant group (29 cases), and the ROC curve was plotted (Figure S1B). The ROC analysis results demonstrated that p53 percentage-score could be used as a valuable biomarker to distinguish *TP53* non-GOF and GOF variants (AUC = 0.960, *p* < 0.001). The Youden Index reached its maximum at p53 percentage-score = 65 (Youden Index = 0.850), with corresponding sensitivity and specificity of 0.897 and 0.953, respectively (Figure S1B).

**Fig. 2:**
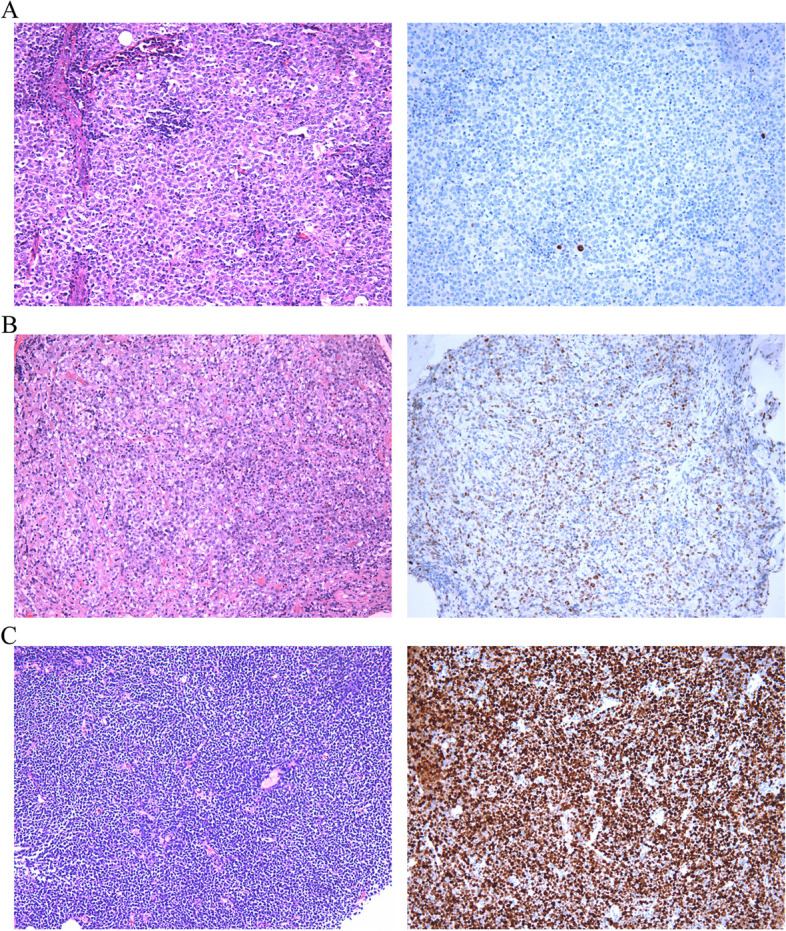
Different patterns of p53 expression in LBCLs. **A**. Representative hematoxylin and eosin (HE) image of Pattern A (left) and representative p53 IHC staining image of Pattern A (right). **B**. Representative HE image of Pattern B (left) and representative p53 IHC staining image of Pattern B (right). **C**. Representative HE image of Pattern C (left) and representative p53 IHC staining image of Pattern C (right)

Thus, those with a p53 percentage-score ≤ 3% were categorized as pattern A, 4%-65% as pattern B, and > 65% as pattern C. Pattern A (Fig. 2A): 0%-3% of tumor cells showed weakly positive staining in a dispersed pattern. Pattern B (Fig. 2B): 4-65% of tumor cells stained positive for p53, and in this pattern, the intensity of p53 IHC staining of tumor cells was mixed, including negative, weakly positive and strongly positive cells. Pattern C (Fig. 2C): More than 65% of tumor cells stained positive for p53. The most common IHC type was type B (50/95, 52.6%), followed by type C (30/95, 31.6%) and type A (15/95, 15.8%).

### Correlation between p53 IHC expression patterns and *TP53* alteration status

P53 IHC expression patterns were associated with *TP53* alterations in LBCLs (*p* < 0.001, Table 2). Among cases with a p53 IHC Pattern B, 92.0% (46/50) were wild-type, and 8.0% (4/50) were mutant/delete type. For the cases of a p53 IHC Pattern A&C, wild-type accounted for 40.0% (6/15) and 10.0% (3/30), while mutant/delete type accounted for 60.0% (9/15) and 90.0% (27/30). Patterns A and C were classified as mutant/delete patterns to predict *TP53* alteration status, whereas pattern B was the wild-type pattern. According to diagnostic tests, the sensitivity, specificity, and overall accuracy of this binary classification approach for *TP53* alteration status prediction were 0.900, 0.836, and 0.863, respectively.

**Table 2:** Comparison between p53 IHC expression patterns and *TP53* alteration status in 95 LBCL cases

IHC staining patterns	Alteration status		*p*-value
	Wild-type	Mutant/delete type	
Pattern types			< 0.001
Pattern A	6	9	
Pattern B	46	4	
Pattern C	3	27	
Binary classification			< 0.001
Wild-type pattern	46	4	
Mutant/delete pattern	9	36	

### Correlation between p53 IHC expression patterns and *TP53* alteration types

Next, we analyzed the relationship between p53 IHC expression patterns and *TP53* alteration types and found a strong correlation (Table 3, *p* < 0.001). Two cases had both LOF and GOF variants, the remaining 93 cases were wild-type or had one type of *TP53* alteration. Two cases with both LOF and GOF variants belong to Pattern A and Pattern C, respectively. Eight cases (88.9%) of LOF variants belonged to the Pattern A group, accounting for 53.3% (8/15) of Pattern A. Among 55 wild-type cases, 46 cases (83.6%) belonged to Pattern B, accounting for 92.0% (46/50) of pattern B. Most of the 29 cases of GOF (93.1%, 27/29) were missense mutations, and 26 cases (89.7%) belonged to Pattern C, accounting for 86.7% of Pattern C. Considering NGS and FISH as the gold standard for *TP53* alterations status detection, we used diagnostic tests to determine the predictive efficacy of Pattern A, Pattern B and Pattern C for LOF variants, wild-type and GOF variants (Table S2). The sensitivity, specificity, and overall accuracy of the three patterns were all higher than 0.800. According to the Kappa consistency analysis, the Kappa-value was 0.730 (*p* < 0.001).

**Table 3:** Comparison between p53 IHC expression patterns and *TP53* alteration types in 95 LBCL cases

IHC staining pattern	Total	Wild type	*TP53* alteration status			*p*-value
			LOF	GOF	Both LOF & GOF	
Pattern A	15	6	8	0	1	< 0.001
Pattern B	50	46	1	3	0	
Pattern C	30	2	0	26	1	
Total	95	55	9	29	2	

### Reproducibility of the p53 IHC expression patterns

**Fig. 3:**
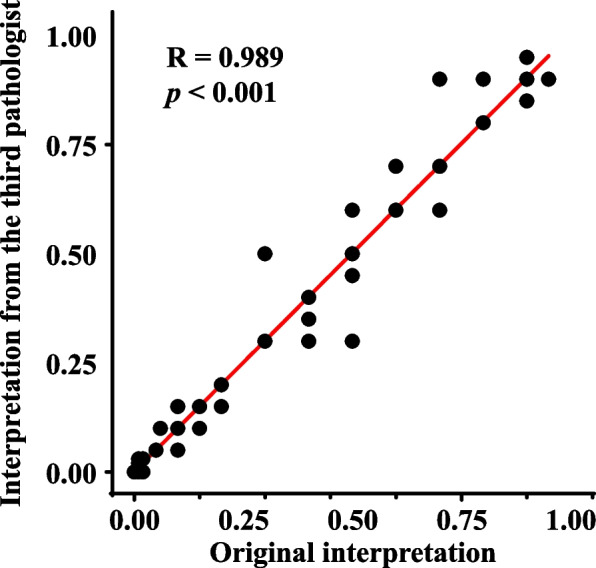
The Pearson analysis showed a strong correlation between the p53 percentage scores by different pathologists (R = 0.989, *p* < 0.001)

A third pathologist was invited to independently assess p53 expression and recorded the p53 percentage-score. According to the Pearson correlation analysis, there was a high correlation between the original interpretation and interpretation from the third pathologist (*R* = 0.989,* p* < 0.001, Fig. 3). The interpretation results of the two groups of pathologists were converted into p53 IHC expression patterns for the Kappa consistency test, and the Kappa value was 0.965 (Table S3).

### Consistency in different LBCL subtypes

LBCLs include 18 specific entities. To test the versatility of p53 IHC expression patterns in LBCLs, we divided LBCLs into 2 groups (DLBCL, NOS, and other subtypes). Among the 95 cases included, there were 75 (78.9%) DLBCL, NOS cases, 20 (21.1%) other subtype cases. No statistically significant differences were shown in age, sex, stage, or alteration type between the two groups (Table S4), and the predictive power of p53 IHC expression patterns was consistent in the two groups (Table S5 and S6).

### p53 H-Score: A more refined approach for classifying p53 expression patterns

Although the p53 percentage-score is widely used in clinical practice and more generalizable, it only considers the proportion of positive cells and overlooks the heterogeneity of p53 expression intensity. Therefore, the p53 H-score was also assessed and statistically analyzed to explore its potential as a classification criterion for p53 IHC expression patterns. ROC curve analysis was utilized to evaluate the discriminative value of p53 H-score for *TP53* LOF variants, wild-type, and GOF variants in LBCLs. The ROC analysis results demonstrated that p53 H-score can serve as a valuable biomarker to discriminate between *TP53* LOF and non-LOF variants, as well as between *TP53* non-GOF and GOF variants. The Youden index reached its maximum at p53 H-score = 3 for LOF variants and p53 H-score = 122.5 for GOF variants (Figure S2).

Thus, those with a p53 H-score less than or equal to 3 were categorized as pattern A, 4-122 as pattern B, and greater than or equal to 123 as pattern C. Next, the relationship between p53 H-Score patterns and *TP53* alteration types was analyzed, revealing a significant correlation (Table S7). Diagnostic tests were employed to determine the predictive performance of Pattern A, Pattern B, and Pattern C for LOF variants, wild-type, and GOF variants (Table S8). All three patterns exhibited high sensitivity, specificity, and overall accuracy, exceeding 0.800. Furthermore, the Kappa consistency analysis demonstrated a Kappa value of 0.746 (*p* < 0.001).

## Discussion

Dysfunction of the *TP53* gene is the basis of the occurrence and development of lymphoproliferative diseases [26, 27], and it also predicts a worse prognosis and curative effect [5]. Therefore, the detection of *TP53* mutations and deletions is of considerable importance in the diagnosis, treatment, and prognosis of LBCL patients. NGS and FISH are currently the main method for detecting *TP53* variants due to the lengthy sequence and diverse variants of *TP53*, but the high cost of the NGS imposes a heavy financial burden on patients. It has become an urgent problem to select a cheap, fast, less invasive, accurate, and reproducible method for the initial screening and prediction of *TP53* alterations in LBCLs. In the present study, to verify whether the p53 IHC expression patterns in LBCLs can be predictors of *TP53* alterations, we analyzed the relationship between the p53 expression patterns and *TP53* alterations in 95 cases.

Previous investigations have demonstrated the association between p53 IHC expression patterns and *TP53* alterations and its potential predictive usefulness in cancer [15, 20]. High p53 expression in mantle cell lymphoma (> 30%) accurately predicts missense mutations, according to Joana M. Rodrigues et al. [28]. Keisuke Sawada et al. revealed that wide p53 expression highly linked with missense mutations in oral epithelial dysplasia. They also validated the strong association between p53 null type and nonsense mutations [29]. Junjie Li et al. found that most cases of p53 loss in gastrointestinal neuroendocrine neoplasms contained LOF variants, whereas weak and strong p53 positive expression tended to have wild-type *TP53* and GOF variants, respectively [15]. Based on previous studies, we obtained similar findings in LBCLs. In our study, the p53 expression patterns were classified according to the p53 percentage-score. Pattern A corresponds to almost no expression of p53, pattern B corresponds to wild-type expression of p53 (scattered mode showing weak positive staining), and pattern C corresponds to overexpression of p53. We confirmed that patterns A, B, and C were strongly correlated with LOF variants, wild-type, and GOF variants, respectively, and could be used as potential predictors of *TP53* alteration types to guide clinical diagnosis and treatment in the future.

Meanwhile, the cutoff value of p53 expression patterns for determining *TP53* alteration status varied between investigations. Joana M. Rodrigues et al. found that p53 expression > 30% predicted *TP53* missense mutations [28]. Anna Yemelyanova et al. observed that ovarian cancer patients with 0% or > 60% p53 expression had *TP53* mutations [20]. In the study of Junjie Li et al., 0% p53 expression was associated with LOF variants, whereas > 60% p53 expression correlated with GOF [15]. In this study, ROC curves were employed to determine the cutoff values for p53 IHC expression patterns, which were found to be 3.5% for predicting *TP53* LOF variants and 65% for GOF variants. Heterogeneity of protein expression, IHC staining variances, and human factors may explain these discrepancies. Heterogeneous protein expression might be caused by uneven protein expression in various tissues and cells. To reduce discrepancies in p53 IHC staining, we documented our platform and antibodies. Considering human factors, a third pathologist was asked to assess p53 staining patterns, and consistency was verified. Different pathologists consistently interpreted the p53 expression patterns, indicating that our study results have strong practicability and therapeutic translational value.

In this study, considering the heterogeneity of p53 expression intensity and extent, the p53 H-score was employed as a second scoring system for pattern classification (p53 H-score pattern A, B, and C), and the results were included in the supplementary materials. The findings of this study demonstrate that similar to the p53 IHC expression pattern based on the p53 percentage-score, the p53 H-score pattern also exhibited excellent performance in predicting the LOF, GOF variations, and wild type of the *TP53* gene. However, the p53 percentage score offers greater convenience in clinical diagnosis, significantly reducing the workload of pathologists and minimizing the biases that may arise from manual scoring. Therefore, considering the p53 percentage-score as a routine evaluation criterion for the p53 IHC expression pattern is more feasible and practical in predicting the variant status of the *TP53* gene in LBCLs. On the other hand, the p53 H-score pattern can be considered as an alternative for future clinical work or utilized in subsequent multicenter large-sample studies to further explore its advantages and disadvantages compared to the p53 percentage-score as a classification criterion for predicting *TP53* alteration types. In addition, with the aim of enabling fellow researchers to replicate our protocols and independently validate our research findings, we have included a comprehensive dataset of the 95 LBCLs used in our study in the supplementary materials (Table S9). This dataset encompasses detailed information, such as pathological diagnosis, p53 IHC interpretation, and *TP53* alteration types. By sharing this source data, we aspire to foster meaningful discussions within the scientific community, gain further insights, and collectively advance our understanding of the classification criteria for *TP53* alterations based on p53 IHC patterns.

Among the 95 LBCL cases we collected, there were two special cases with both LOF and GOF variants. The p53 expression patterns of these two cases were pattern B and pattern C, respectively. Due to the limitation of the number of cases, the p53 expression patterns of these two special cases could not be explained in this study. We hypothesized that when missense mutations and nonsense mutations coexist, the p53 expression patterns may be related to the alteration sites of *TP53*, and the specific mechanism needs to be further studied.

We also investigated the *TP53* mutation locations and the single nucleotide substitutions. The majority (75%) of *TP53* mutations in cancers are missense mutations in the DNA-binding core domain (codons 100-300) [30]. We confirmed that the majority of *TP53* mutations in LBCLs are indeed located in the DNA-binding core domain, and most of them are located in exon 7. Although codons 175 and 273 are the most common *TP53* mutations in human tumors [30], the most frequent *TP53* mutation codon in DLBCL is 248 [31]. Our statistical results showed that 248 was indeed the codon with the highest mutation frequency among 95 LBCLs. Of all the single nucleotide variants, the C > T mutation accounted for the highest proportion of 43.6%, indicating the unique mutation preference of LBCLs.

LBCLs belong to a large family, but there exists some heterogeneity between different entities. We divided DLBCLs into one group, and the other types of entities were divided into another group for analysis. There was no characteristic difference between DLBCLs and non-DLBCL LBCLs, and the relationship between the p53 expression patterns and *TP53* alterations remained consistent with previous conclusions. However, with in-depth research, we can further discuss the relationship and mechanism between the p53 expression patterns and *TP53* alterations in specific LBCL entities in the future.

The strength of this study is that our center has a relatively large number of LBCL samples for analysis. All cases were reviewed by experienced pathologists to ensure the accuracy of diagnosis. Our study also has some limitations. We collected subjects from only one hospital, which may have led to selection bias. This limitation may be addressed in the future by sharing data with other clinical organizations.

## Conclusions

**Fig. 4:**
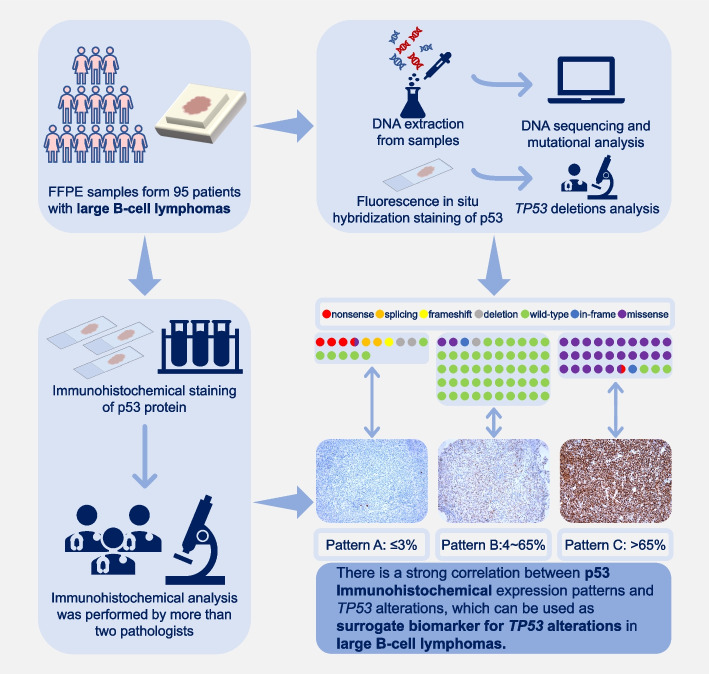
Schematic diagram of research ideas and results

In conclusion, our work validates the predictive value of p53 IHC expression patterns for *TP53* alteration status and types in LBCLs, demonstrating that p53 expression patterns can be used as practical and efficient surrogate biomarkers for *TP53* alterations (Fig. 4). Our findings provide new alternative ideas for medical institutions in underdeveloped regions with imperfect molecular detection platforms and provide opportunities to reduce the financial burden on patients and reduce the medical system strain.

### Supplementary Information

**Additional file 1:** **Table S1.** Large B-cell lymphomas of WHO Classification of Hematolymphoid Tumors, 5th edition: B-cell lymphoid proliferations and lymphomas. **Figure S1.** A. Receiver operating characteristic (ROC) curves displaying the sensitivity and specificity of p53 percentage-score for discriminating loss of function (LOF) and non-LOF variants in *TP53*. Insets indicate area under curve (AUC) values, 95% confidence intervals and statistics. The optimal cut-off points, determined by the Youden Index, are denoted by green dots. B. ROC curves displaying the sensitivity and specificity of p53 percentage-score for discriminating non-gain of function (GOF) and GOF variants in *TP53*. Insets indicate AUC values, 95% confidence intervals and statistics. The optimal cut-off points, determined by the Youden Index, are denoted by green dots. **Table S2.** Diagnostic tests and Kappa identity test results for p53 expression patterns A, B and C. **Table S3.** The interpathologist concordance for p53 immunohistochemical (IHC) patterns in large B-cell lymphomas. **Table S4.** Clinicopathological characteristics of 75 diffuse large B-cell lymphomas and 20 other large B-cell lymphomas. **Table S5.** Comparison between p53 IHC patterns and *TP53 *alteration types in 75 diffuse large B-cell lymphomas. **Table S6.** Comparison between p53 IHC patterns and *TP53 *alteration types in 20 other large B-cell lymphomas. **Figure S2.** A. ROC curves displaying the sensitivity and specificity of p53 H-score for discriminating LOF and non-LOF variants in *TP53*. Insets indicate AUC values, 95% confidence intervals and statistics. The optimal cut-off points, determined by the Youden Index, are denoted by green dots. B. ROC curves displaying the sensitivity and specificity of p53 H-score for discriminating non-GOF and GOF variants in *TP53*. Insets indicate AUC values, 95% confidence intervals and statistics. The optimal cut-off points, determined by the Youden Index, are denoted by green dots. **Table S7.** Comparison between p53 H-Score patterns and *TP53 *alteration types in 95 LBCL cases. **Table S8.** Diagnostic tests and Kappa identity test results for p53 H-Score pattern A, B and C. **Table S9.** Source data of the 95 LBCLs.

## Data Availability

The datasets generated and/or analyzed during the current study, which focused on the *TP53* gene alterations in large B-cell lymphomas, do not fall under the categories of data types that require deposition in external repositories as they do not constitute high-throughput sequencing data. In the supplementary materials, we have provided the raw data necessary for the analysis of p53 immunohistochemistry patterns and *TP53* alterations. Researchers interested in accessing and verifying the results presented in this study may contact the corresponding author for further information and data availability, subject to compliance with ethical and legal requirements regarding patient privacy and data protection. The corresponding author will make reasonable efforts to accommodate such requests, ensuring adherence to applicable regulations and guidelines.

## Abbreviations

LBCL: Large B-cell lymphoma
DLBCL: Diffuse large B-cell lymphoma
NOS: Not otherwise specified
IHC: Immunohistochemistry
GOF: Gain of function
LOF: Loss of function
FFPE: Formalin fixed paraffin-embedded
VAF: Variant allele frequency
NGS: Next generation sequencing
FISH: Fluorescence in situ hybridization
ROC: Receiver operating characteristic
AUC: Area under curve
