# Supplementary material for: Accurate interpretation of p53 immunohistochemical patterns is a surrogate biomarker for TP53 alterations in large B-cell lymphoma
Source: BMC Cancer. 2023 Oct 19;23:1008. doi: 10.1186/s12885-023-11513-x (PMC10588220; doi:10.1186/s12885-023-11513-x)
Supplement: Supplementary file 1 — Additional file 1: Table S1. Large B-cell lymphomas of WHO Classification of Hematolymphoid Tumors, 5th edition: B-cell lymphoid proliferations and lymphomas. Figure S1. A. Receiver operating characteristic (ROC) curves displaying the sensitivity and specificity of p53 percentage-score for discriminating loss of function (LOF) and non-LOF variants in TP53. Insets indicate area under curve (AUC) values, 95% confidence intervals and statistics. The optimal cut-off points, determined by the Youden Index, are denoted by green dots. B. ROC curves displaying the sensitivity and specificity of p53 percentage-score for discriminating non-gain of function (GOF) and GOF variants in TP53. Insets indicate AUC values, 95% confidence intervals and statistics. The optimal cut-off points, determined by the Youden Index, are denoted by green dots. Table S2. Diagnostic tests and Kappa identity test results for p53 expression patterns A, B and C. Table S3. The interpathologist concordance for p53 immunohistochemical (IHC) patterns in large B-cell lymphomas. Table S4. Clinicopathological characteristics of 75 diffuse large B-cell lymphomas and 20 other large B-cell lymphomas. Table S5. Comparison between p53 IHC patterns and TP53 alteration types in 75 diffuse large B-cell lymphomas. Table S6. Comparison between p53 IHC patterns and TP53 alteration types in 20 other large B-cell lymphomas. Figure S2. A. ROC curves displaying the sensitivity and specificity of p53 H-score for discriminating LOF and non-LOF variants in TP53. Insets indicate AUC values, 95% confidence intervals and statistics. The optimal cut-off points, determined by the Youden Index, are denoted by green dots. B. ROC curves displaying the sensitivity and specificity of p53 H-score for discriminating non-GOF and GOF variants in TP53. Insets indicate AUC values, 95% confidence intervals and statistics. The optimal cut-off points, determined by the Youden Index, are denoted by green dots. Table S7. Comparison between p53 H [file 12885_2023_11513_MOESM1_ESM.pdf]

Supplementary Information

Accurate interpretation of p53 immunohistochemical patterns is a surrogate biomarker for *TP53* alterations in large B-cell lymphoma

CONTENTS

Table S1 2

Figure S1 3

Table S2 4

Table S3 5

Table S4 6

Table S5 7

Table S6 8

Figure S2 9

Table S7 10

Table S8 11

Table S9 12

Table S1: Large B-cell lymphomas of WHO Classification of Hematolymphoid Tumors, 5th edition: B-cell lymphoid proliferations and lymphomas.

Large B-cell lymphomas
Diffuse large B-cell lymphoma, NOS
T-cell/histiocyte-rich large B-cell lymphoma
Diffuse large B-cell lymphoma/ high grade B-cell lymphoma with MYC and BCL2 rearrangements
ALK-positive large B-cell lymphoma
Large B-cell lymphoma with IRF4 rearrangement
High-grade B-cell lymphoma with 11q aberrations
Lymphomatoid granulomatosis
EBV-positive diffuse large B-cell lymphoma
Diffuse large B-cell lymphoma associated with chronic inflammation
Fibrin-associated large B-cell lymphoma
Fluid overload-associated large B-cell lymphoma
Plasmablastic lymphoma
Primary large B-cell lymphoma of immune-privileged sites
Primary cutaneous diffuse large B-cell lymphoma, leg type
Intravascular large B-cell lymphoma
Primary mediastinal large B-cell lymphoma
Mediastinal grey zone lymphoma
High-grade B-cell lymphoma, NOS

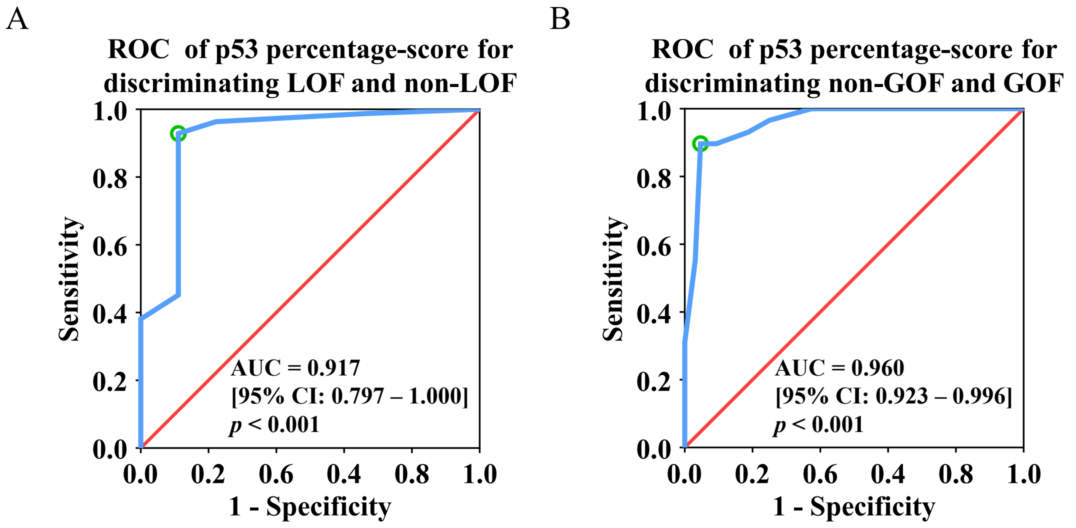

Figure S1: A. Receiver operating characteristic (ROC) curves displaying the sensitivity and specificity of p53 percentage-score for discriminating loss of function (LOF) and non-LOF variants in *TP53*. Insets indicate area under curve (AUC) values, 95% confidence intervals and statistics. The optimal cut-off points, determined by the Youden Index, are denoted by green dots. B. ROC curves displaying the sensitivity and specificity of p53 percentage-score for discriminating non-gain of function (GOF) and GOF variants in *TP53*. Insets indicate AUC values, 95% confidence intervals and statistics. The optimal cut-off points, determined by the Youden Index, are denoted by green dots.

Table S2: Diagnostic tests and Kappa identity test results for p53 expression patterns A, B and C.

Diagnostic tests and kappa identity test*	Pattern A	Pattern B	Pattern C
Sensitivity	0.889	0.836	0.897
Specificity	0.919	0.900	0.939
Overall accuracy	0.811	0.863	0.926
Kappa value		0.730	

*Next generation sequencing (NGS) and fluorescence in situ hybridization (FISH) analysis were used as the gold standard for the detection of p53 IHC expression patterns as surrogate biomarkers for *TP53* alterations in large B-cell lymphoma.

Table S3: The interpathologist concordance for p53 immunohistochemical (IHC) patterns in large B-cell lymphomas.

Original interpretation	Interpretation from the third pathologist		
	Pattern A	Pattern B	Pattern C
Pattern A	15	0	0
Pattern B	0	49	1
Pattern C	0	1	29

Table S4: Clinicopathological characteristics of 75 diffuse large B-cell lymphomas and 20 other large B-cell lymphomas.

Characteristics	Histological type		Total number	*p*-Value
	Others (n = 20)	DLBCL (n = 75)		
Gender				0.811
male	11 (55.0)	39 (52.0)	50 (52.6)	
female	9 (45.0)	36 (48.0)	45 (47.4)	
Age (year)				0.243
≤ 35	7 (35.0)	9 (12.0)	16 (16.8)	
36 - 45	1 (5.0)	9 (12.0)	10 (10.5)	
46 - 55	3 (15.0)	16 (21.3)	19 (20.0)	
56 - 65	4 (20.0)	21 (28.0)	25 (26.3)	
> 65	5 (25.0)	20 (26.7)	25 (26.3)	
Stage				0.295
Not applicable/ Difficult to determine	4 (20.0)	6 (8.0)	10 (10.5)	
I	2 (10.0)	8 (10.7)	10 (10.5)	
II	5 (25.0)	13 (17.3)	18 (18.9)	
III	0 (0.0)	8 (10.7)	8 (8.4)	
IV	9 (45.0)	40 (53.3)	49 (51.6)	
Alteration types				0.844
Wild type	12 (60.0)	43 (57.3)	55 (57.9)	
LOF	1 (5.0)	8 (10.7)	9 (9.5)	
GOF	7 (35.0)	22 (29.3)	29 (30.5)	
Both LOF & GOF	0 (0.0)	2 (2.7)	2 (2.1)	

DLBCL: diffuse large B-cell lymphomas

Table S5: Comparison between p53 IHC patterns and *TP53* alteration types in 75 diffuse large B-cell lymphomas.

IHC staining pattern	Total	Wild type	*TP53* alteration status			*p*-value
			LOF	GOF	Both LOF & GOF	
Pattern A	13	5	7	0	1	< 0.001
Pattern B	41	37	1	3	0	
Pattern C	21	1	0	19	1	
Total	75	43	8	22	2	

Table S6: Comparison between p53 IHC patterns and *TP53* alteration types in 20 other large B-cell lymphomas.

IHC staining pattern	Total	Wild type	*TP53* alteration status			*p*-value
			LOF	GOF	Both LOF & GOF	
Pattern A	2	1	1	0	0	< 0.001
Pattern B	9	9	0	0	0	
Pattern C	9	2	0	7	0	
Total	20	12	1	7	0	

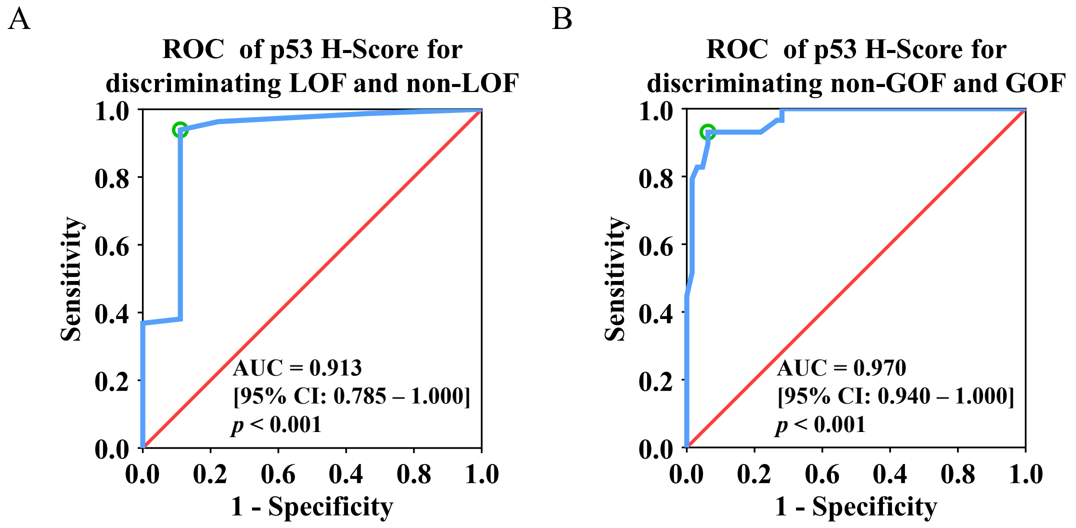

Figure S2: A. ROC curves displaying the sensitivity and specificity of p53 H-score for discriminating LOF and non-LOF variants in *TP53*. Insets indicate AUC values, 95% confidence intervals and statistics. The optimal cut-off points, determined by the Youden Index, are denoted by green dots. B. ROC curves displaying the sensitivity and specificity of p53 H-score for discriminating non-GOF and GOF variants in *TP53*. Insets indicate AUC values, 95% confidence intervals and statistics. The optimal cut-off points, determined by the Youden Index, are denoted by green dots.

Table S7: Comparison between p53 H-Score patterns and *TP53* alteration types in 95 LBCL cases.

IHC staining pattern	Total	Wild type	*TP53* alteration status			*p*-value
			LOF	GOF	Both LOF & GOF	
Pattern A	13	5	8	0	0	< 0.001
Pattern B	50	46	1	2	1	
Pattern C	32	4	0	27	1	
Total	95	55	9	29	2	

Table S8: Diagnostic tests and Kappa identity test results for p53 H-Score pattern A, B and C.

Diagnostic tests and kappa identity test*	Pattern A	Pattern B	Pattern C
Sensitivity	0.889	0.836	0.931
Specificity	0.942	0.900	0.924
Overall accuracy	0.947	0.863	0.926
Kappa value		0.746	

*Next generation sequencing (NGS) and fluorescence in situ hybridization (FISH) analysis were used as the gold standard for the detection of p53 H-Score patterns as surrogate biomarkers for *TP53* alterations in large B-cell lymphoma.

Table S9: Source data of the 95 LBCLs.

ID	Pathological diagnosis	p53 IHC interpretation			*TP53* alteration type
		Strong (%)	Moderate (%)	Weak (%)	
1	non-DLBCL LBCL	0	0	0	splicing
2	DLBCL	0	0	0	wild-type
3	DLBCL	0	0	0	nonsense
4	DLBCL	0	0	0	nonsense
5	DLBCL	0	0	1	deletion
6	DLBCL	0	0	1	wild-type
7	DLBCL	0	0	1	wild-type
8	DLBCL	0	0	1	splicing
9	DLBCL	0	0	1	frameshift
10	DLBCL	0	0	1	nonsense
11	DLBCL	0	0	2	wild-type
12	non-DLBCL LBCL	0	0	2	wild-type
13	DLBCL	0	2	0	wild-type
14	DLBCL	0	2	0	nonsense+missense
15	DLBCL	0	0	2	deletion
16	DLBCL	0	0	5	wild-type
17	DLBCL	0	0	5	wild-type
18	DLBCL	0	2	3	wild-type
19	DLBCL	0	2	3	wild-type
20	DLBCL	0	1	4	wild-type
21	non-DLBCL LBCL	0	0	5	wild-type
22	non-DLBCL LBCL	2	4	0	wild-type
23	DLBCL	0	0	10	wild-type
24	DLBCL	0	2	8	wild-type
25	DLBCL	0	2	8	wild-type
26	DLBCL	0	10	0	wild-type
27	DLBCL	0	0	10	wild-type
28	DLBCL	0	0	10	wild-type
29	DLBCL	5	0	15	wild-type
30	DLBCL	0	10	0	wild-type
31	DLBCL	0	0	10	wild-type
32	non-DLBCL LBCL	0	2	8	wild-type
33	DLBCL	0	0	15	wild-type
34	DLBCL	0	5	10	wild-type
35	non-DLBCL LBCL	0	0	15	wild-type
36	non-DLBCL LBCL	0	5	10	wild-type
37	DLBCL	0	10	10	wild-type
38	DLBCL	0	20	0	wild-type
39	non-DLBCL LBCL	0	0	20	wild-type
40	non-DLBCL LBCL	0	5	15	wild-type

41	DLBCL	0	0	20	wild-type
42	non-DLBCL LBCL	15	15	0	wild-type
43	DLBCL	0	30	0	wild-type
44	DLBCL	0	0	30	wild-type
45	DLBCL	0	0	30	wild-type
46	DLBCL	2	20	8	missense
47	non-DLBCL LBCL	0	10	20	wild-type
48	DLBCL	10	10	10	wild-type
49	DLBCL	3	7	20	wild-type
50	DLBCL	3	20	7	wild-type
51	DLBCL	10	30	0	wild-type
52	DLBCL	20	20	0	wild-type
53	DLBCL	0	40	0	wild-type
54	DLBCL	5	10	25	in-frame
55	DLBCL	0	40	0	wild-type
56	DLBCL	25	25	0	missense
57	DLBCL	0	0	50	wild-type
58	DLBCL	0	0	50	wild-type
59	DLBCL	10	40	0	wild-type
60	DLBCL	10	40	0	wild-type
61	DLBCL	20	30	0	wild-type
62	DLBCL	20	30	0	deletion
63	DLBCL	0	0	60	wild-type
64	DLBCL	5	20	35	wild-type
65	DLBCL	40	20	0	wild-type
66	DLBCL	20	50	0	missense
67	DLBCL	70	0	0	missense
68	DLBCL	70	0	0	in-frame
69	DLBCL	70	0	0	missense
70	DLBCL	20	50	0	missense
71	non-DLBCL LBCL	30	40	0	wild-type
72	non-DLBCL LBCL	70	0	0	missense
73	DLBCL	70	0	0	missense
74	DLBCL	70	0	0	missense
75	DLBCL	70	0	0	missense
76	non-DLBCL LBCL	50	20		missense
77	DLBCL	60	20	0	wild-type
78	DLBCL	30	50	0	missense
79	DLBCL	80	0	0	missense
80	DLBCL	80	0	0	missense
81	non-DLBCL LBCL	80	0	0	missense
82	DLBCL	80	0	0	missense
83	DLBCL	50	30	0	missense
84	non-DLBCL LBCL	60	20	0	wild-type

85	DLBCL	60	20	0	nonsense+missense
86	non-DLBCL LBCL	60	20	0	missense
87	DLBCL	90	0	0	missense
88	non-DLBCL LBCL	90	0	0	missense
89	DLBCL	90	0	0	missense
90	DLBCL	90	0	0	missense
91	DLBCL	90	0	0	missense
92	DLBCL	70	30	0	missense
93	non-DLBCL LBCL	90	0	0	missense
94	non-DLBCL LBCL	90	0	0	missense
95	DLBCL	95	0	0	missense

DLBCL: diffuse large B-cell lymphomas; LBCL: Large B-cell lymphoma; IHC: Immunohistochemistry.
